# Barth Syndrome Cardiomyopathy: An Update

**DOI:** 10.3390/genes13040656

**Published:** 2022-04-08

**Authors:** Jing Pang, Yutong Bao, Kalia Mitchell-Silbaugh, Jennifer Veevers, Xi Fang

**Affiliations:** 1 Department of Medicine, University of California San Diego, La Jolla, CA 92093, USA; jpang@ucsd.edu (J.P.); ybao@ucsd.edu (Y.B.); kmitchel@ucsd.edu (K.M.-S.); jenveevers@gmail.com (J.V.); 2 Department of Biological Science, University of California San Diego, La Jolla, CA 92093, USA

**Keywords:** Barth syndrome, cardiomyopathy, TAFAZZIN, cardiolipin

## Abstract

Barth syndrome (BTHS) is an X-linked mitochondrial lipid disorder caused by mutations in the *TAFAZZIN* (*TAZ*) gene, which encodes a mitochondrial acyltransferase/transacylase required for cardiolipin (CL) biosynthesis. Cardiomyopathy is a major clinical feature of BTHS. During the past four decades, we have witnessed many landmark discoveries that have led to a greater understanding of clinical features of BTHS cardiomyopathy and their molecular basis, as well as the therapeutic targets for this disease. Recently published *Taz* knockout mouse models provide useful experimental models for studying BTHS cardiomyopathy and testing potential therapeutic approaches. This review aims to summarize key findings of the clinical features, molecular mechanisms, and potential therapeutic approaches for BTHS cardiomyopathy, with particular emphasis on the most recent studies.

## 1. Introduction

Barth syndrome (BTHS) is a rare, X-linked genetic disorder caused by mutations in the *TAFAZZIN* (also known as *G4.5*, formerly annotated as *TAZ*) gene on chromosome Xq28 [1,2,3,4]. The estimated prevalence of BTHS is approximately one case in every million births, occurring primarily within the male population [5]. The clinical features include cardiomyopathy, skeletal myopathy, neutropenia, growth retardation, and recurrent infections [4]. Cardiomyopathy is the major clinical feature diagnosed in 70% of patients within their first year of life [4,6]. Patients with severe cardiomyopathy are subjected to heart transplantation [4,6]. However, the current treatment of BTHS cardiomyopathy mainly aims at symptom reduction.

BTHS cardiomyopathy presents key features of mitochondrial cardiomyopathy illustrated by mitochondrial malformations and dysfunction in the cardiac tissues of BTHS patients [1,4,6]. The major pathognomonic metabolic abnormality in BTHS is the elevation of the monolysocardiolipin (MLCL) to cardiolipin (CL) ratio [7,8,9,10,11], ushering the critical function of TAZ in CL remodeling. Defective CL has been shown to significantly impair the structure and function of mitochondria [12]. The defect in CL remodeling has been specifically linked to BTHS [7,8,9,10,11], thus underscoring the critical role of TAZ-mediated CL remodeling in cardiac mitochondria and cardiomyopathy. Clinical reports reveal an increasing survival rate in BTHS patients in recent decades, and those who survive through infancy can live into their late forties with stabilized cardiac function [13]. These findings highlight the importance of studying detailed molecular mechanisms and identifying the potential therapeutic targets of BTHS cardiomyopathy. Recently published *Taz* knockout mouse models provided an unequivocal model to investigate the molecular basis for BTHS cardiomyopathy and test potential therapeutic approaches [14,15]. In this review, we will focus on the clinical features, molecular mechanisms, and potential therapeutic approaches of BTHS cardiomyopathy, including recent updates.

## 2. TAZ, CL and BTHS

**Figure 1:**
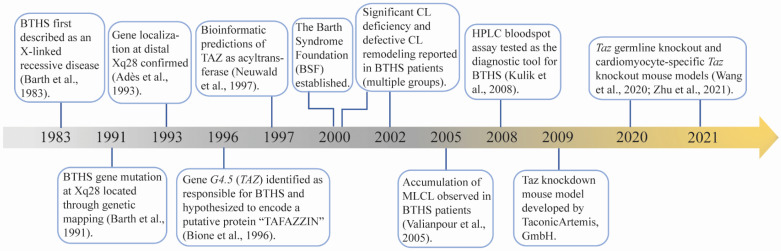
Major discoveries of Barth syndrome (BTHS) cardiomyopathy. Key advancements in the investigation of BTHS from 1983 until now [1,2,3,9,10,14,15,16,20]. CL: cardiolipin; MLCL: monolysocardiolipin; HPLC: high-performance liquid chromatography.

BTHS was first described by Dr. Peter Barth in 1983 as an X-linked mitochondrial disease that affects cardiac muscle, skeletal muscle, and neutrophil leukocytes [1]. In the following four decades, intensive investigations have been performed to understand the molecular basis and clinical features of BTHS (Figure 1). In 1991, the disease locus of BTHS was mapped to a gene-rich region in the distal portion of Xq28 [2]. Five years later, mutations in *G4.5* were identified as responsible for BTHS [3]. The *G4.5* encoded protein was named TAFAZZIN (TAZ) after a masochistic television character “Tafazzi” that was popular in Italy when Bione et al. cloned this gene [3]. TAZ was found to be highly expressed in cardiac and skeletal muscle [3]. Following bioinformatic prediction that TAZ belongs to a superfamily consisting of acyltransferases involved in phospholipid biosynthesis and/or remodeling [16], Vreken et al. reported that the CL levels were significantly decreased in cultured skin fibroblasts derived from BTHS patients, although the biosynthesis rates of CL and its processor phosphatidylglycerol (PG) were normal [8]. Moreover, the remodeling of PG and CL, particularly the incorporation of linoleic acid into PG and CL, was disturbed in BTHS patient-derived fibroblasts [8]. Subsequently, the abnormalities not only included a reduced CL concentration and altered fatty acyl side chain [7,8,17,18], but also an elevated level of MLCL, an intermediate product of CL remodeling [9]. Later on, specific characteristics of the CL profile, including low total CL concentration, abnormal CL fatty acyl composition, and elevated MLCL to CL ratio, became distinguishing biochemical defects in BTHS patients [7,8,9,10,11,18]. These CL abnormalities serve as unique and distinct features of BTHS. Because MLCL levels are typically low in healthy individuals, an elevated MLCL to CL ratio allows for a sensitive and definitive diagnosis of BTHS [4,11]. However, there is limited availability for this testing in a clinical setting. Increased 3-methylglutaconic acid (3-MGCA) in blood and urine, a direct byproduct of aberrant aerobic energy metabolism [19], has been observed in BTHS patients. Although 3-MGCA levels are highly variable in patients and apply to several other mitochondrial and non-mitochondrial disorders [4], the presence of high 3-MGCA levels in the blood and/or urine of an individual with cardiomyopathy and/or neutropenia should significantly raise the suspicion for BTHS. Further genetic testing for *TAZ* mutation is used to definitively confirm BTHS [4].

More than 200 pathogenic mutations in *TAZ* have been reported to cause BTHS. The Human *TAZ* gene variants database is available on the Barth Syndrome Foundation (BSF) website (https://www.barthsyndrome.org/research/tafazzindatabase.html, accessed on 14 October 2020). These mutations are spread across all 11 exons of *TAZ*, resulting in the complete absence or decreased levels of TAZ protein, or loss of TAZ protein function. However, no consistent genotype/phenotype correlations have been demonstrated so far. Because of the trace amount of MLCL present in healthy individuals [11], any increase in the MLCL level could be observed as a dramatic increase. There is no correlation between the MLCL/CL ratio and genotype or disease phenotype.

The function of TAZ as acyltransferase or transacylase was first hypothesized based on the abnormal CL profile in BTHS patients [7,8,9,17,18], and later supported by biochemical studies [21,22]. Deletion of the *Taz* gene in yeasts [23,24] and drosophila [25] further demonstrated the function of TAZ. However, the results from in vitro experiments showed that TAZ reversibly reacted with a broad spectrum of phospholipids without any intrinsic acyl specificity [26,27], which fails to explain why it selectively transfers particular acyl groups (e.g., linoleoyl group) into CL in vivo. Some studies suggest that the physical properties, such as non-bilayer-type lipid domains [28], and/or the crowded protein environment, such as large amounts of mitochondrial respiratory chain complexes [29], of the mitochondrial membrane might create conditions that promote TAZ enzymatic activity and substrate specificity. TAZ is localized in mitochondria and associated with mitochondrial membranes [30,31,32]. However, the precise sub-mitochondrial localization of TAZ remains ambiguous. TAZ protein has been shown to be assembled into multiple protein complexes [30,33,34]. The biological consequence of these interactions remains unclear, although one report suggested that the assembly of TAZ into protein complexes could be critical for its stability [35].

**Figure 2:**
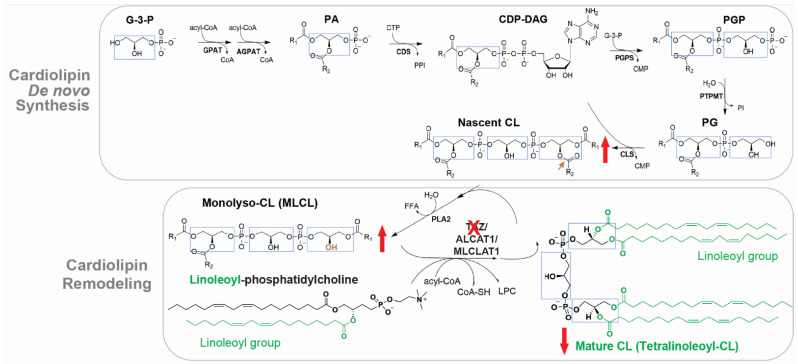
Cardiolipin (CL) de novo synthesis and remodeling pathways. Glyceraldehyde 3-phosphate (G-3-P) is catalyzed by a series of enzymes to generate nascent CL. To achieve a final symmetric acyl composition (e.g., TetralinoleoyI-CL in the heart), nascent CL undergoes an extensive remodeling process by deacylation to monolyso-CL (MLCL), and subsequent reacylation catalyzed by specific acyltransferases including Tafazzin (TAZ). GPAT: glycerol-3-phosphate acyltransferase; AGPAT: 1-acylglycerol-3-phosphate-O-acyltransferase; PA: phosphatidic acid; CDS: cytidine diphosphate diacylglycerol synthases; CDP-DAG: cytidine diphosphate-diacylglycerol; PGP: phosphatidylglycerol phosphate; PG: phosphatidylglycerol; PGPS: PGP synthase; PTPMT1: protein tyrosine phosphatase mitochondrial 1; CLS: CL synthase; PLA2: phospholipase A2; MLCL: monolyso-CL; PC: phosphatidylcholine; LPC: lysophosphocholine; ALCAT1: acyl-CoA:lysocardiolipin acyltransferase 1; MLCLAT1: monolysocardiolipin acyltransferase 1. Light blue box: glycerol backbone; Red-down arrows (↓): decreased in BTHS; Red-up arrow (↑): increased in BTHS; Green content: Linoleoyl groups; Red cross (X): TAZ loss of function in BTHS.

CL is exclusively associated with mitochondrial membranes and is primarily localized to the inner mitochondrial membrane (IMM) where it contributes up to 20% of total lipids [12]. CL is composed of a glycerol head group and two PG backbones bound by four fatty acyl chains, the composition of which determines its biophysical properties [12,22]. In the mammalian heart, the predominant form of CL is tetralinoleoyl-CL, which contains four linoleic acid moieties (C18:2) (Figure 2). CL de novo synthesis occurs exclusively in the IMM via a process that is highly conserved from yeasts to mammals [22]. Briefly, CL synthesis is initiated upon the formation of phosphatidic acid (PA) and is catalyzed by a series of enzymes to produce nascent CL (also known as unremodeled CL) [22] (Figure 2). Because enzymes of the de novo CL synthesis pathway lack acyl specificity, nascent CL contains a mixture of fatty acyl chains that differ in length and saturation [22]. The formation of the unique acyl composition of mature CL is derived from a remodeling process in which acyl chains are removed and replaced with other acyl chains [21,22]. In the human heart, tetralinoleoyl-CL accounts for around 80% of CL acyl chains [36,37], suggesting the extensive remodeling in the heart. The major model of CL remodeling is a deacylation/reacylation process [21,22]. However, this model is challenged by the transacylation mechanism, by which acyl groups shuttle between ester bonds without deacylation [29,38,39]. In the two-step deacylation/reacylation process (Figure 2), nascent CL is first deacylated to MLCL by phospholipase A2 (PLA2) [21,22]. Subsequently, the acyl group from acyl-CoA or other IMM phospholipids (e.g., linoleoyl-phosphatidylcholine) is transferred to MLCL by acyltransferase or transacylase activity, forming a remodeled fatty acyl chain [22]. CL is fully remodeled to achieve the final symmetric acyl composition via several cycles of deacylation/reacylation [22]. Mammals have at least three enzymes capable of re-acylating MLCL, including TAZ [38], acyl-CoA:lysocardiolipin acyltransferase-1 (ALCAT1) [40], and MLCL acyltransferase 1 (MLCLAT1) [41]. TAZ mutations lead to abnormal CL profile in BTHS, including low total CL concentration, abnormal CL fatty acyl composition, and elevated MLCL to CL ratio (Figure 2, red arrows).

## 3. Clinical Features of BTHS Cardiomyopathy

Cardiomyopathy and heart failure are the primary reasons for diagnosis and death in BTHS [4,42]. According to the report from the Barth Syndrome Registry in 2012 [43], 70% of BTHS patients were recognized as having cardiomyopathy in the first year of life and 12% required cardiac transplantation. Recent data showed that the median age at transplant was 1.7 years [42], in agreement with the well-acknowledged perspective that infancy is a high-risk age group [4,42]. However, some intercurrent viral infections cause myocarditis-like symptoms which mask the BTHS cardiomyopathy, leading to a delay in the genetic or metabolic diagnosis of BTHS [4,42].

The most common cardiomyopathy presented in BTHS is dilated cardiomyopathy (DCM), characterized by the weakening of the heart muscle and enlarged ventricles [4,42]. Additionally, BTHS patients sometimes present left ventricular noncompaction, and less frequently have hypertrophic and restrictive cardiomyopathy [4,42]. Other cardiac issues include arrhythmia, prolonged corrected QT interval, endocardial fibroelastosis, sudden cardiac arrest, and fetal cardiomyopathy with or without intrauterine fetal demise [4]. The cardiomyopathy in BTHS patients is often presented in a mixed and/or fluctuating fashion [42,43,44]. Cardiac phenotypes of patients with BTHS mainly affect the left ventricle and rarely affect the right ventricle [45]. Similar to other clinical findings in BTHS, the cardiomyopathy phenotype is widely variable in presentation, morphology, response to therapy, and long-term outcome [42].

As an X-linked genetic disease, BTHS occurs almost exclusively in males [4]. In familial BTHS, female carriers are usually asymptomatic [4]. It is theoretically possible for a female to manifest symptoms of BTHS due to skewed X-inactivation. For example, one female BTHS patient has been described as having mosaicism for monosomy X and for a ring X chromosome with a large deletion of the long arm including the Xq28 region [46]. This patient experienced severe heart failure at one month of age and was diagnosed with BTHS, along with dilated-hypokinetic and hypertrophic cardiomyopathy with left ventricular noncompaction [46].

Histopathological studies on heart biopsies from BTHS patients revealed endomyocardial fibroelastosis, interstitial fibrosis, and myocyte hypertrophy [46,47,48]. Electron microscopic studies revealed increased numbers of enlarged and roughly spherical mitochondria, combined with circular and tubular mitochondrial cristae [4,48,49,50]. Vacuolated myocytes and lipid microvesicles were also observed in BTHS hearts [4,49]. 

Although BTHS is a multisystem disorder, cardiomyopathy plays a major role in the outcomes and progression of BTHS [4,42]. Data obtained from longitudinal studies provide an overview of BTHS cardiomyopathy [43,44,51,52]. Infancy and early childhood are high-risk periods for cardiac death and transplant in BTHS [43,44,51]. However, cardiac function is frequently stabilized or even normalized throughout adolescence and into adulthood [52]. After stabilization, although most of the patients are still on cardiac medication(s), their left ventricle size is in the upper normal range, and their systolic function is low–normal or mildly depressed [42]. In patients who present a stabilized cardiac function after adolescence, cardiomyopathy becomes a more benign symptom, or even subclinical [52]. There are also patients who exhibit a lower response to therapy, or who respond well initially but deteriorate after months or years of stability, necessitating cardiac transplantation [44,48,51,52]. Detailed mechanisms of those with progressive heart failure, compared to those who demonstrate stabilization of cardiac function, have not been elucidated. Notably, the survival rate of BTHS is increasing in recent decades [13,42]. Affected individuals who were born before the year 2000 had a five-year survival rate of 22%, compared to a survival rate of 70% in those born in or after the year 2000 [13]. This recent increase in the survival rate is probably due to improvements in disease diagnosis and management, thus emphasizing the importance of early recognition as well as the development of effective therapies to treat the disease at different stages [13,42]. 

## 4. Experimental Models of BTHS Cardiomyopathy

The initial studies utilized fibroblasts derived from BTHS patients, including patient-derived lymphoblasts [8] and skin fibroblasts [8,11]. Following the initial discovery that *TAZ* mutation causes BTHS, multiple experimental models of BTHS have been established by genetic silencing or manipulation of the *TAZ* gene, including yeast, drosophila, zebrafish, cell, and mouse [53]. These experimental models recapture some aspects of BTHS molecular and/or pathophysiological phenotypes. Studies from these models significantly improve our understanding of TAZ function and BTHS, and are invaluable for testing therapeutic approaches for BTHS. Recently published *Taz* knockout mouse models [14,15], which recapture pathophysiological phenotypes of BTHS, have significantly advanced research on BTHS. The major model systems of BTHS have been reviewed recently [53]. Here, we will specifically focus on the experimental models of BTHS cardiomyopathy, including Taz knockdown (KD) mice, *Taz* global knockout (gKO) mice, and *Taz* cardiomyocyte-specific knockout (cKO) mice, as well as mouse embryonic stem cell (ESC)-derived cardiomyocytes, and human-induced pluripotent stem cell (iPSC)-derived cardiomyocytes (Table 1).

### 4.1. Taz KD Mouse Model

A major difficulty in generating *Taz* Knockout (KO) mice is obtaining germline transmission from Male chimeras carrying the *Taz* KO allele [72], probably due to defects in Male germ cell meiosis and spermatogenesis [72,73,74] that render male chimeras infertile [72]. Thus, a doxycycline-inducible short hairpin RNA (shRNA) mediated Taz KD mouse model (https://www.jax.org/strain/014648, accessed on 1 November 2021) was established by TaconicArtemis, GmbH (Köln, Germany) under contract with BSF, and became the most widely used mouse model in BTHS research [54,75]. The genetic information of Taz KD mice has been described in detail [54,65]. In this Tet-On inducible shRNA expression system, doxycycline was administered in drinking water [67,76], chow [54,56,57,58,59,60,61,62,63,64,65,66,68,77], or water and chow in combination [55], to induce KD of Taz. Although the doxycycline induced Tet-On system provides the convenience of reversible Taz KD following the withdrawal of doxycycline, the continuous and high dose doxycycline treatment potentially impacts mitochondrial function and metalloprotease activity [75]. Thus, it is important to consider the effect of doxycycline alone when analyzing the phenotypes and molecular mechanisms in doxycycline-induced Taz KD mice. 

Doxycycline administration achieved the effective KD of Taz, although the different administration strategies resulted in variable results (Table 1). The administration in chow at 200 or 625 mg/kg throughout the gestation and postnatal life resulted in a 90% or 97% reduction of *Taz* mRNA, respectively, in the heart at 2 months of age [54,57,58,59,60,61,63,65]. Although the accumulation of MLCL and a shift toward more saturated CL species has been observed in the tafazzin-deficient hearts, these Taz KD mice developed normally and exhibited similar body weight as littermate controls until 8 months of age [54,63], in contrast to the early onset phenotypes presented in BTHS patients [4,43]. Mild DCM phenotypes and abnormalities in mitochondria morphology and ultrastructure were reported in Taz KD mice at 7–8 months of age [54,55,58,65]. However, the cardiac function and mitochondria morphology were normal in Taz KD heart at 2 months of age, whereas the mitochondrial respiration capacity was decreased [57,59]. Detailed mitochondrial abnormalities in Taz KO hearts and the underlying mechanisms are discussed in Section 5.1, Section 5.2 and Section 5.3. Adrenergic stress induced by chronic isoproterenol treatment at 4.5 months of age exacerbated the cardiac dysfunction in Taz KD mice [55]. Interestingly, a recent study reported that Taz KD mice developed heart failure with preserved ejection fraction and an age-dependent progression of diastolic dysfunction in the absence of fibrosis starting at 10 weeks of age [63]. Administration of doxycycline (625 mg/kg in chow) at 2 months of age established a mouse model with acquired deficiency of TAZ in the adult heart [68]. The induction of Taz KD at the adult stage resulted in more than 80% decreased *Taz* mRNA levels and a 40% reduction in tetralinoleoyl-CL [68]. Although the increased reactive oxygen species (ROS) and increased susceptibility to permeability transition pore (mPTP) opening were observed in the “acquired” Taz KD mice, the respiration rate was unchanged [68]. The authors also found that the susceptibility to ex vivo ischemia-reperfusion injury was comparable between Taz KD and control hearts [68]. 

Although the Taz KD resulted in typical BTHS CL abnormalities, the phenotypes were not as severe as BTHS patients, which was puzzling as TAZ deficiency and abnormal CL metabolism are widely recognized as pathological causes of the disease phenotype. However, the levels of TAZ protein were not reported in most of the studies [65,68], probably due to limited access to specific TAZ antibodies as noted [65,68]. On the other hand, although the elevated MLCL level has been found in all the reports, there was no sufficient analysis on the absolute levels of total CL, which includes remodeled and unremodeled CL species, or the levels of each CL species. Because of the trace amount of MLCL present in healthy cells [11], any increase in the MLCL level could be observed as a dramatic increase. However, the elevated MLCL alone may not result in disease phenotype [9]. A certain level of decrease in the total CL, mature CL level, or in combination, could be the key pathogenetic factor. The residual functional TAZ protein may be able to catalyze some CL remodeling and meet the demand at early stages. Although it has not been identified, it is also possible that the residual functional TAZ protein may serve different functional role(s), independent of CL remodeling, sufficient for mice survival and normal cardiac function at early stages. In addition, some unpublished data suggested that the genetic backgrounds of the model mice could be a factor in variable phenotypes [53]. Understanding how Taz KD mice preserve basal cardiac function despite persistent CL abnormalities by careful comparisons of the full parameter CL profile and the molecular pathways between *Taz* KD and KO models could reveal key molecular mechanisms that result in BTHS, shedding light on novel strategies for attenuating the development of cardiomyopathy in BTHS.

While most studies show relatively mild cardiac phenotypes [54,55,56,57,58,60,61,62,63,64,65,75], one report showed striking cardiac dysfunction including noncompaction cardiomyopathy and heart failure at embryonic and neonatal stages [76]. In this study, 2 mg/mL doxycycline dose in 10% sucrose drinking water was administered to the pregnant females on the day of plug. In estimation, the mice in this study ingested 3–10 times more doxycycline than 200 or 625 mg/kg in chow [75,76]. In embryos, 80% KD of *Taz* mRNA could be achieved within three days, and a BTHS typical CL pattern, as well as abnormal mitochondria morphology, was observed in Taz KD mice [76]. Given the phenotypes of these Taz KD mice were much more severe than the *Taz* gKO and cKO mice (see Section 4.2), the high dose of doxycycline was considered as a major factor, in addition to TAZ deficiency, causing the striking phenotype [53]. Notably, the severe phenotype was not reported when the mice were treated with the same dose of doxycycline in drinking water starting at 3 months of age [67].

### 4.2. Taz gKO and cKO Mouse Models

Recently, *Taz* floxed mouse models were generated by two independent groups utilizing the traditional ES cell gene targeting method [14] and clustered regularly interspaced short palindromic repeats (CRISPR)/CRISPR-associated protein 9 (Cas9) technology [15], respectively. In both mouse models, exons 5 to 10 of the *Taz* gene were flanked by 2 LoxP sites [14,15]. The *Taz* floxed mice were maintained in a C57BL6/J [14] or C57BL/6NCrl [15] background and were used to generate *Taz* gKO and cKO mice.

The *Taz* null allele was generated by germline Cre-mediated recombination [14,73]. Heterozygous females (*Taz* ^null/+^) were crossed with wildtype male mice to generate hemizygous *Taz* gKO male mice (*Taz* ^null/Y^) and their control littermates (*Taz* ^+/Y^) [14]. Capillary immunoblotting of cardiac tissue confirmed the completed deletion of TAZ protein. As expected, the MLCL to CL ratio was elevated in the *Taz* gKO hearts [14]. *Taz* gKO mice were born below the expected Mendelian ratio; however, whether the *Taz* gKO mice displayed an embryonic phenotype remains unknown [14]. About 80% of the *Taz* gKO mice died at their neonatal stages with lower body weight than control littermates. At postnatal day (P)1, *Taz* gKO mice were observed with a mild to moderate decrease in left ventricular systolic function compared to controls [14]. About 20% of *Taz* gKO mice with an initial body weight of more than 1.2 g at P1 displayed better survival, although they remained smaller than control littermates throughout life [14]. Although it remains unknown how long the postneonatal survivors can live, the 120-day survival curve of *Taz* gKO mice mirrors the life expectancy of BTHS patients, as many affected BTHS patients die in infancy or early childhood, and those who live into adulthood can survive into their late forties [13]. The *Taz* gKO survivors displayed DCM with cardiomyocyte apoptosis and increased cardiac fibrosis starting at 3 months of age. A greater number of mitochondria were found with abnormal spatial distribution in *Taz* gKO myocardium. The *Taz* gKO mitochondria were smaller, displaying simplified and disorganized cristae. Notably, abnormal sarcomere structure was also reported in *Taz* gKO hearts [14]. Taken together, *Taz* gKO recapitulates the disease phenotype of BTHS patients, providing a true *Taz* null mouse model for BTHS research.

The perinatal lethality and systematic defects of *Taz* gKO mice precluded the study of the specific role of *Taz* and CL remodeling in adult heart function. Tissue-specific deletion of *Taz* can be achieved by crossing floxed mice with Cre mouse lines that express Cre recombinase under the control of a tissue-specific promoter or enhancer. Hemizygous *Taz* cKO male (*Taz^F^*^/Y^; Cre+) mice were generated by utilizing two widely used cardiomyocyte-specific Cre mice lines, myosin heavy chain 6 (Myh6)-Cre (*Taz* cKO:*Myh6*-Cre) [14,69] and *Xenopus laevis* myosin light-chain 2 (Xmlc2)-Cre (*Taz* cKO:*Xmlc2*-Cre) [15], respectively. Effective deletions of *Taz* in cKO hearts have been confirmed in both *Taz* cKO mouse models [14,15]. Besides the MLCL/CL ratio that has been assessed in most of the BTHS experimental models [14,18,23,35,54,55,65,74,76,78,79], Zhu et al. reported a full parameter analysis of CL profiles, including an assessment of CL and MLCL levels, as well as the fatty acyl side-chain composition of CL in ventricular tissues isolated from *Taz* cKO and control mice [15]. The results revealed a 50% reduction in total CL levels in cKO hearts and a 50-fold increased MLCL/CL ratio in *Taz* cKO hearts. The accumulations of unremodeled CL with shorter or more saturated chains and decreased mature CL levels, as well as the accumulations of precursors and intermediate products of CL biosynthesis, demonstrated the inefficient CL remodeling in *Taz* cKO hearts [15]. Both *Taz* cKO:*Myh6*-Cre [14] and *Taz* cKO:*Xmlc2*-Cre [15] mice were born at expected Mendelian ratios and survived through the perinatal stages, suggesting that perinatal lethality is not due to the loss of TAZ in cardiomyocytes. The cardiac phenotypes were reported as slightly different between *Taz* cKO:*Myh6*-Cre [14] and *Taz* cKO:*Xmlc2*-Cre [15] mice. Wang et al. reported that *Taz* cKO:*Myh6*-Cre mice had normal cardiac function at 1 month of age, but the cardiac function progressively declined starting at 2 months of age. The cardiac dysfunction is slightly more aggressive than observed in *Taz* gKO mice, and is accompanied by an increase in left ventricular end-diastolic diameter (LVEDD), as well as an elevated heart weight normalized to body weight ratio [14]. Zhu et al. analyzed a large group of *Taz* cKO:*Xmlc2*-Cre mice and found that a small fraction (fewer than 5%) of *Taz* cKO:*Xmlc2*-Cre mice exhibited lethality before 2 months of age with significantly enlarged hearts, whereas the majority of *Taz* cKO:*Xmlc2*-Cre mice survived until 50 weeks of age [15]. The surviving *Taz* cKO:*Xmlc2*-Cre mice displayed ventricular dilation and compromised heart function at 4 months, although cardiac morphology and function were not altered at 2 months of age [15]. In contrast to the *Taz* cKO:*Myh6*-Cre mice that displayed rapidly and progressively declined cardiac function [14], the surviving *Taz* cKO:*Xmlc2*-Cre mice displayed impaired but stable cardiac function [15], consistent with recent findings in surviving patients with BTHS [13]. Notably, *Taz* cKO:*Xmlc2*-Cre mice did not display overtly enlarged hearts or an increased heart weight/body weight ratio, as typically seen in other DCM models, although the end-diastolic LV internal diameter (LVIDd) was significantly increased [15]. At 4 months of age, the hearts of surviving *Taz* cKO:*Xmlc2*-Cre mice appeared to have a more rounded shape relative to control littermates [15]. Zhu et al. did not observe any cardiac arrhythmias in *Taz* cKO:*Xmlc2*-Cre mice by surface electrocardiogram (ECG) at 2 weeks, 2 months, and 6 months of age [15]. However, Liu et al. reported arrhythmias in *Taz* cKO:*Myh6*-Cre mice under intracardiac electrophysiology study at 6 weeks of age, when they had mild or no cardiac dysfunction [69]. Long-term telemetric ECG recordings of electrograms in ambulatory mice are important to evaluate whether *Taz* cKO mice display cardiac arrhythmias. Moreover, myocardial fibrosis and cardiomyocyte apoptosis has been observed in *Taz* cKO:*Myh6*-Cre hearts [14], but not in *Taz* cKO:*Xmlc2*-Cre hearts [15]. Detailed mitochondrial analyses before overt cardiac dysfunction revealed mitochondrial malformations and dysfunction in *Taz* cKO:*Xmlc2*-Cre hearts (detailed in sections below) [15], further demonstrating that the *Taz* cKO mouse model mirrors multiple physiological and biochemical aspects of BTHS cardiomyopathy. Validation of the fidelity of the *Taz* gKO and cKO mouse models for studies of BTHS cardiomyopathy paves the way for our further understanding of the cause of BTHS cardiomyopathy and for testing future therapies for BTHS cardiomyopathy. It is worth mentioning that Wang and Pu noted a strain background that could significantly affect the survival and the progression of cardiomyopathy in *Taz* gKO mice (unpublished result) [53], suggesting the involvement of strong genetic modifiers. Identification of these genetic modifiers may lead to novel therapeutic strategies to treat BTHS and may explain the high variability in the clinical presentation of BTHS.

### 4.3. Mouse ESC-Derived Cardiomyocytes and Human iPSC-Derived Cardiomyocytes

Pluripotent stem cells, including ESC and iPSC-derived cardiomyocytes, offer an attractive experimental platform to model cardiovascular diseases in the culture dish. Stem cell-derived cardiomyocytes share characteristics and functional properties of primary cardiomyocytes, although they are more similar to fetal or neonatal cardiomyocytes and lack some properties of adult cardiomyocytes [80]. A *Taz* KO ESC model was generated by Acehan et al. and subsequently induced into cardiomyocytes [70]. Analysis of the mitochondria ultrastructure revealed that lamellar-type mitochondria were less abundant in differentiated *Taz* KO cardiomyocytes. The aberrant cristae in differentiated *Taz* KO cardiomyocytes lost their parallel orientation and formed branching lamellae, suggesting that Taz deficiency inhibited the mitochondrial differentiation [70]. However, the defects in undifferentiated *Taz* KO ESC were minor, although both ESC and ESC-derived cardiomyocytes displayed BTHS CL defects.

Human iPSC-derived cardiomyocytes are much closer to human hearts than other systems, and thus provide an unprecedented opportunity to study the molecular mechanisms underlying cardiomyopathy caused by TAZ deficiency and to test potential interventions for BTHS cardiomyopathy in human cardiomyocytes. The iPSC-derived cardiomyocyte models for BTHS have been generated from BTHS patient-derived [57,71] and genetically engineered iPSCs carrying *TAZ* mutations [71]. Not surprisingly, BTHS iPSC-derived cardiomyocytes displayed abnormal CL profiles with elevated MLCL to CL ratios [57,71]. BTHS iPSC-derived cardiomyocytes had similar mitochondrial numbers compared to the control iPSC-derived cardiomyocytes, but the size of mitochondria in BTHS iPSC-derived cardiomyocytes was smaller than controls [71]. Impaired mitochondrial respiration capacity [57,71] and elevated ROS have been observed in BTHS iPSC-derived cardiomyocytes. Interestingly, BTHS iPSC-derived cardiomyocytes exhibit abnormal sarcomerogenesis [57,71] and impaired contractility [71]. Gene replacement and genome editing demonstrated that *TAZ* mutation is necessary and sufficient for the disease phenotypes [71].

## 5. Delineating the Molecular Mechanism of BTHS Cardiomyopathy

**Figure 3:**
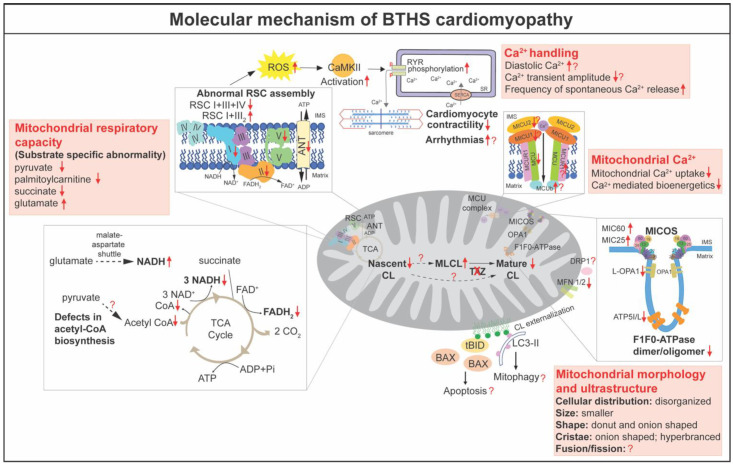
The molecular basis of BTHS cardiomyopathy. Defective TAZ and abnormal CL in BTHS result in numerous abnormalities in mitochondrial bioenergetics, morphogenesis, and architecture, as well as ROS and calcium (Ca^2+^) homeostasis. The question marks denote the unknown or controversial aspects. RYR: ryanodine receptors; MCU: mitochondrial Ca^2+^ uniporter; MICU: mitochondrial calcium uptake proteins; MICOS: mitochondrial contact site and cristae organizing system; TCA: tricarboxylic acid cycle; ANT: adenine nucleotide translocator; ROS: reactive oxygen species; Red-down arrows (↓): down-regulation; Red-up arrow (↑): up-regulation; Red question marks (?): uncertainty as to the molecular mechanism in BTHS cardiomyopathy.

CL is essential for numerous mitochondrial functions, including bioenergetics, oxidative stress, membrane architecture and organization, fusion and fission, mitophagy, iron homeostasis, and the regulation of apoptosis [12]. Defective TAZ and abnormal CL metabolism have been shown to result in mitochondrial abnormalities. In this section, we will discuss the molecular mechanism of BTHS cardiomyopathy in detail (Figure 3). 

### 5.1. Mitochondrial Bioenergetics

Mitochondria are the major source of cellular energy in the heart, producing ATP via the respiratory chain in the IMM, with the tricarboxylic acid cycle (TCA cycle, also called the Krebs or citric acid cycle) products NADH and FADH2 delivering electrons to respiratory complexes I and II, respectively [81]. The lower respiratory rates in skeletal muscle mitochondria isolated from the first described BTHS patients became the initial evidence to define BTHS as a mitochondrial disorder [1]. Thus far, the reduced mitochondrial respiration capacity has been reported in multiple BTHS patient-derived cell lines, including fibroblasts [82], lymphoblasts [83,84], and iPSCs [57,71]. Similar defects have been observed in the BTHS cardiomyocytes and tissues [57,59,60,62,63,71]. Unexpectedly, two reports of iPSC-derived cardiomyocytes revealed increased basal respirations, probably due to compensatory mechanisms, while the maximal respiration capacity was severely impaired in these BTHS iPSC-derived cardiomyocytes in contrast to the controls [57,71]. Consequently, the ATP levels in BTHS iPSC-derived cardiomyocytes were significantly lower than those in controls cultured in a galactose-based medium, which limited ATP production via glycolysis [71]. Experiments with the Taz KD mice showed reduced respiration capacity in the mitochondria [57,59,60,62,63] and neonatal cardiomyocytes [62] isolated from the Taz KD hearts when pyruvate, palmitoylcarnitine, or succinate was provided as substrates. Recent data from *Taz* cKO hearts further confirmed the reduction of respiration capacity in the TAZ deficient mitochondria at 2 months of age before cardiac dysfunction [15]. These results demonstrated the essential role of TAZ in mediating CL remodeling in mitochondrial respiration. Here, we discuss the molecular mechanism by which TAZ deficiency impairs mitochondrial bioenergetics from different regulatory aspects of mitochondrial respiration.

The mitochondrial respiratory chain, also called the electron transport chain, is at the center of mitochondrial bioenergetics. The respiratory chain consists of four large protein complexes (I–IV), as well as F1F0 ATP synthase (complex V) embedded in the IMM [85]. The first angle to dissect the mechanism, by which TAZ deficiency causes the reduction of mitochondrial respiration capacity, is analyzing the protein level and activity of the individual respiratory complexes. However, the results are variable. In cardiac tissues from BTHS patients, the activity of complex I was decreased [86]. Inefficient complex V activity was attributed to the reduced ATP generation in BTHS patient iPSC-derived cardiomyocytes [71]. In Taz KD mice, the activities of complex I [59,64], complex II [57,64], III [57,60,62,64], complex IV [59,64], and/or complex V [60] were decreased in the heart. However, recent data from the mitochondria isolated from *Taz* cKO heart revealed no alteration in the enzymatic activity of individual complexes I, II, III, or IV [15]. Proteomics analysis of Taz KD hearts revealed that the NDUFA5 subunit of complex I, the catalytic SDHA subunit of complex II, Rieske (UQCRFS1), cytochrome c1 (CYC1) subunits of complex III, and ATP5A1, the catalytic core-forming subunit ATPA of complex V, were decreased in TAZ deficient hearts [61]. 

For efficient electron transport to occur, respiratory chain complexes must be assembled into large oligomers of different compositions and stoichiometry, referred to as respiratory chain supercomplexes (RCS) [81]. RCS are formed by complex I, which builds a platform for binding of dimeric complex III (III_2_) and several copies of complex IV [81]. It has been suggested that RCS are destabilized in lymphoblasts [83,87], fibroblasts [82], and iPSC derived from patients with BTHS [88], as well as in *Taz* KO in yeast [31]. Although these studies achieve a consensus that TAZ deficiency results in a more labile supercomplex containing complexes I, III_2_, and IV, it is controversial as to whether TAZ deficiency destabilizes individual complexes or low molecular weight heterooligomeric complexes. Cultured lymphoblasts from BTHS patients showed decreased amounts of supercomplexes containing I and III_2_ [83,87], as well as the individual complexes I, III, and IV [87]. However, analysis of the mitochondria isolated from *Taz* KD and cKO hearts revealed that the heterooligomeric forms of complex I and III_2_ and the individual complexes were increased in *Taz* KD or cKO mitochondria, whereas amounts of high molecular weight RCS containing complex I, III_2_, and IV were significantly decreased [15,57,63], consistent with results from BTHS patient-derived fibroblasts [82] and iPSC [88]. Notably, the results from *Taz* cKO mitochondria pointed out that loss of TAZ impairs the bridging of complex IV to RCS because the amount of RCS that contains complex IV was diminished, but heterooligomeric complexes without complex IV were accumulated in *Taz* cKO mitochondria [15]. The destabilized RCS might result from the loss of interaction between CL and respiratory chain complex proteins. It has been proposed that CL, which is intimately associated with all of the respiratory chain complexes, acts as a crucial glue that stabilizes the assembly of individual respiratory chain complexes into RCS [89,90,91,92]. On the other hand, the assembly of respiratory chain complexes triggers CL remodeling [29]. 

In mitochondrial oxidative phosphorylation (OXPHOS), NADH and FADH_2_ generated by the TCA cycle are oxidized back to NAD^+^ and FAD^+^, respectively, providing electrons that funnel into the respiratory chain. In a normal mitochondrion, NADH and FADH2 are maintained at low levels due to the high efficiency of the respiratory chain. The rising concentration of NADH that results from decreased respiration capacity subsequently inhibits TCA cycle enzymes [81]. In BTHS patient-derived lymphoblasts, the ratio of NAD+/NADH is significantly decreased, implying a potential inhibition of TCA cycle enzymes [87]. Surprisingly, a stable isotope tracer-based metabolomics approach revealed that, in BTHS lymphoblasts, TCA cycle metabolites and enzyme activities were clearly not affected when glucose, oleic acid, or glutamine was provided as substrate respectively [82]. A recent study in *Taz* KD mice demonstrated that the levels of free CoA and acyl-CoA content were dramatically lower in the hearts from *Taz* KD, compared to wildtype controls [59], in agreement with the role of CL in acetyl-CoA biosynthesis discovered in yeast [93]. In the same study, the authors found an upregulation in respiration capacity in *Taz* KD hearts when glutamate (amino acid) was used as the substrate, while the respiration capacity was decreased in *Taz* KD hearts when pyruvate or palmitoylcarnitine was used as the substrate [59], consistent with previous publications [60,61]. Importantly, the upregulation of mitochondrial respiration supported by glutamate oxidation in *Taz* KD hearts reaches respiration rates comparable to those achieved with pyruvate and palmitoylcarnitine in wildtype mice. This suggests that TAZ deficiency does not intrinsically impair cardiac mitochondrial respiratory capacity, but rather selectively impairs the oxidation of fatty acids and pyruvate and induces a compensatory increase in glutamate oxidation capacity [59]. Incubation of *Taz* KD mitochondria with exogenous CoA partially rescued pyruvate and palmitoylcarnitine oxidation capacities. Given that pyruvate and palmitoylcarnitine both require CoA to support OXPHOS in mitochondria, whereas glutamate can be oxidized to generate NADH by CoA-independent reactions via the malate-aspartate shuttle, the authors concluded that dysregulation of CoA-dependent intermediary metabolism rather than respiratory chain defects impacts the bioenergetics in TAZ deficiency [59]. 

The adenine nucleotide translocator (ANT, also called ADP/ATP carrier) has been known to mediate the exchange of ADP and ATP across the IMM [94]. CL tightly binds to ANT and maintains its conformation [95,96,97,98]. The molecular composition of CL has been shown to modulate ANT activity in a substrate-specific manner to direct bioenergetic metabolite oxidation [99]. Consistently, analysis of functional ANT activity revealed a selective increase in glutamate-stimulated activity in isolated cardiac mitochondria from Taz KD mice, whereas the ANT activity was not altered in other substrates [60]. Moreover, the decreased abundance of large protein assemblies containing the ANT was observed in Taz KD mouse tissues and BTHS lymphoblasts [39]. However, how these defects in ANT contribute to the cardiac phenotype of BTHS has not been elucidated. 

### 5.2. Reactive Oxygen Species (ROS), Calcium (Ca^2+^) Homeostasis, and Apoptosis

Mitochondria are a major source of ROS, which are a byproduct of mitochondrial electron transfer activity. Reduction in the activity of respiratory chain complexes could result in elevated levels of ROS, including superoxide [81]. Most of studies in BTHS iPSC-derived cardiomyocytes [57,69,71], Taz KD [57,58,59,69], and KO cardiac mitochondria [53,69] reported elevated levels of ROS. However, Goncalves et al. reported that superoxide production was indistinguishable between Taz KD and control hearts [100]. The contractility of BTHS iPSC-derived cardiomyocytes was rescued by a mitochondrially targeted ROS scavenger MitoTEMPO, but not by culture conditions that normalized ATP levels, suggesting that elevated ROS generation participates in the pathogenesis of contractile dysfunction [71]. However, overexpressing catalase in Taz KD mice does not prevent cardiac dysfunction observed at 7–8 months of age [58], suggesting that resolving oxidative stress is not sufficient to suppress BTHS cardiomyopathy.

Ca^2+^ transient plays a central role in cardiomyocyte excitation–contraction coupling. Liu et al. reported increased diastolic Ca^2+^ and decreased Ca^2+^ transient amplitude in both BTHS iPSC-derived cardiomyocytes and *Taz* KO cardiomyocytes [69]. In *Taz* KO cardiomyocytes, excessive ROS activated Ca^2+^/calmodulin-dependent protein kinase II (CaMKII), which increased phosphorylation of RYR2, the main cardiomyocyte intracellular Ca^2+^ release channel, resulting in elevated diastolic Ca^2+^, reduced Ca^2+^ transient amplitude, and increased frequency of spontaneous Ca^2+^ release events [69]. Notably, ROS scavenger MitoTEMPO and CaMKII inhibitor AIP partially rescued the Ca^2+^ handling in BTHS iPSC-derived cardiomyocytes and *Taz* KO cardiomyocytes [69]. Recently, Bertero et al. assessed the contractility and Ca^2+^ transients in Taz KD cardiomyocytes [63], and found that Taz KD cardiomyocytes displayed enhanced fractional sarcomere shortening compared with wildtypes. While the amplitude of Ca^2+^ transients was unchanged at baseline, the rate of Ca^2+^ decay was faster in the Taz KD cardiomyocytes [63]. Under β-adrenergic agonist isoproterenol and 5 Hz stimulation, they found that the accumulation of diastolic Ca^2+^ was lower in Taz KD [63]. 

Mitochondrial Ca^2+^ is thought to play an important role in the regulation of cardiac bioenergetics and function [81]. Mitochondrial Ca^2+^ uptake is tightly controlled by the mitochondrial Ca^2+^ uniporter (MCU) complex in the IMM [81]. Ghosh et al. showed reduced abundance and activity of endogenous MCU in BTHS patient-derived lymphocytes and cardiac tissues [101], as well as in *Taz* KO C2C12 cells [102]. By utilizing the yeast surrogate system, Ghosh et al. demonstrated the specific requirement of CL for MCU stability and activity, as well as mitochondrial calcium transport [101]. Recently, Ghosh et al. reported that, similar to the MCU, the abundance and stability of mitochondrial calcium uptake proteins 1 and 2 (MICU1 and MICU2) were reduced in *Taz* KO C2C12 cells, BTHS patient-derived lymphocytes, and cardiac tissues, whereas the protein levels of EMRE and MCU regulator 1 (MCUR1) were not affected [102]. Furthermore, MCU-complex-dependent mitochondrial Ca^2+^ uptake and Ca^2+^-mediated mitochondrial bioenergetics were disrupted in Taz-KO C2C12 cells [102]. Consistently, Bertero et al. reported that Ca^2+^ uptake was absent in Taz KD mitochondria. This defect is unaffected by cyclosporine A, a mitochondrial permeability transition pore (mPTP) inhibitor, ruling out the possibility of premature mPTP opening [63]. Although decreased MCU protein was observed in Taz -KD hearts, in contrast to the previous report [102], Bertero et al. found that the dominant-negative MCU β- subunit (MCUb) and MCUR1 were increased, but MICU1 and MICU2 were not changed in Taz-KD hearts at 10 weeks of age [63]. They also noted that, unlike cardiac mitochondria, the Ca^2+^ uptake is preserved in skeletal muscle and brain mitochondria of Taz KD mice despite modest changes in MCU levels, although the tissue-specific regulatory mechanism remains unclear [63]. Thus far, the expressions of mitochondrial Ca^2+^ transport machinery and the mitochondria Ca^2+^ transient in the in vivo *Taz* KO model have not been reported yet. On the other hand, Ca^2+^ has been shown to induce CL transition to a non-bilayer state in vitro, and the tetralinoleoyl CL-formed nanodisks are likely susceptible to Ca^2+^-induced non-bilayer transition [37]. However, the interaction between Ca^2+^ and CL in cardiomyocyte remains unknown.

Mitochondria play a key role in apoptotic responses. The externalization of CL into the outer mitochondrial membrane (OMM) forms a binding platform for the recruitment of multi-protein complexes, which are required for the execution of apoptosis [22]. The externalized CL has been suggested to serve as a receptor to recruit tBid to the mitochondrial membrane [103] and is required for Bax activation and OMM permeabilization [104]. CL deficiency significantly impairs tBid binding to the mitochondria [103]. CL is also essential for the recruitment, oligomerization, and activation of caspase-8 on the OMM [87,105]. BTHS-derived lymphoblasts and TAZ KD Hela cells resist the induction of apoptosis [105]. However, it remains unknown whether this is the case in vivo. It is controversial whether apoptosis is increased in TAZ deficient cardiomyocytes [14,15]. 

### 5.3. Morphogenesis, Dynamics and Mitophagy

Mature rod-shaped cardiomyocytes contain thousands of end-to-end contacted spheroid mitochondria, which are densely confined among myofibrils to ensure quick and efficient energy fluxes [81]. The functions of mitochondria are intimately linked to specific mitochondrial characteristics, including a complex double-membrane architecture and specialized cristae structure, continuous fusion and fission, and a quality control mechanism (mitophagy) [81]. Abnormalities in mitochondrial morphology were observed with electron microscopy analysis on heart biopsies from the first few incidences of BTHS in the 1970s and 80s, even before the identification of the *TAZ* mutation [1,48]. It is noted that heart muscle displayed more severe mitochondrial morphological defects than skeletal muscle in BTHS patients [48]. The malformations of cardiac mitochondria in BTHS include disorganized distribution, variable size and shape, onion-shaped cristae, and the accumulation of dense bodies [1,48]. The abnormal mitochondrial morphology and ultrastructure have been reported in multiple models of BTHS, including BTHS patient-derived cells [71,84,106], Taz deletion yeast [23,33,35,79], Taz KD [54,55,58,65], and KO mouse models [14,15]. BTHS iPSC-derived cardiomyocytes displayed greater mitochondrial fragmentation compared to the more highly networked mitochondria of controls [71]. In Taz KD hearts, although the majority of mitochondria were normal in size and structure, they frequently presented in large aggregates between sarcomeres [54]. Multiple concentric layers of densely packed cristae with onion-shaped morphology were common and may indicate that mitochondria were undergoing degeneration or mitophagy. Some mitochondria also contained patches of swollen tubular cristae and large vacuoles at 7–8 months of age [54,55,58,65]. Recent studies revealed abnormal cellular organization, morphology, and ultrastructure of mitochondria in *Taz* gKO [14] and cKO hearts [15]. Similar to the observation in human BTHS hearts, *Taz* cKO hearts displayed variable sizes and abnormal shapes of mitochondria, disorganized and hyperbranched cristae, and a significantly increased number of onion-shaped and donut-shaped mitochondria at 2 months of age, prior to the evident cardiac dysfunction [15]. Detailed quantification showed the increased numbers, reduced area, and increased length of mitochondria in *Taz* cKO hearts, suggesting that some mitochondria became elongated with a smaller cross-section [15]. Zhu et al. further applied a machine learning approach and sphere fitting algorithm to determine mitochondrial size and complexity, and found that cKO hearts displayed smaller but more heterogeneous mitochondrial shapes [15].

CL localizes in highly bent regions within the cristae structures, such as the contact sites [12]. TAZ-mediated CL remodeling is proposed to reshuffle acyl groups between CL and adjacent membrane lipids and to create tightly packed membrane curvature [28]. “Onion-shaped” mitochondria, which have been observed in the hearts of BTHS patients and mouse models, are typical manifestations of defects in the IMM and cristae architecture [107,108]. The structural organization of the IMM and the cristae structures are formed by the mitochondrial contact site and cristae organizing system (MICOS), located at the cristae junctions. Deletion of MICOS components in both yeast [109,110] and mammalian cells [111,112] results in the loss of cristae junctions and the appearance of “onion-shaped” cristae, similar to those observed in BTHS hearts [1,4,48,78]. Recently, Zhu et al. reported that MIC60 and MIC25 were dramatically increased in the proteins isolated from *Taz* cKO hearts and those increased MICOS components were incorporated into the whole MICOS complex [15]. This result is consistent with the finding in BTHS skin fibroblasts [82]. Notably, CL deficiency does not result in the increase of MICOS [113], suggesting that the increased MICOS is probably not due to the decreased CL levels. TAZ has been shown to localize to the contact sites and dynamically interact with protein complexes [30]. Interestingly, deletion of MICOS subunits depletes mitochondrial cristae and decreases TAZ protein, resulting in CL profiles similar to those of TAZ-deficient mutants [114]. This result suggests that synergistic functions occur between TAZ and MICOS at the cristae junction. It remains unknown whether loss of TAZ protein itself disturbs the formation of the cristae junction, or if the defects are dependent on the fatty acid side chain composition of CL or the elevated MLCL levels. Besides MICOS, deletion of Taz in cardiomyocytes results in the reduction of ATP5I and ATP5L, two subunits essential F1F0-ATPase complexes for the cristae tip formation [109]. Furthermore, dimeric and oligomeric F1F0-ATPase complexes are decreased in *Taz* cKO hearts [15]. Notably, overexpression of MIC60 leads to reduced levels of F1F0-ATPase oligomers, enlargement of cristae junction diameters, and branching of cristae in yeast, as observed in *Taz* cKO hearts [15,109]. Thus, the decreased dimerization and oligomerization of F1F0-ATPase complexes could be a result of the combination of decreased ATF5I and ATP5L and/or increased MIC60 and MIC25. However, the role of TAZ and/or CL in regulating the protein levels of ATP5I and ATP5L remains unknown. In addition to MICOS and F1F0-ATP synthase complexes, some other CL binding proteins, such as prohibitin (PHB) family proteins [115,116,117] and optic atrophy 1 (OPA1) [116,118,119], affect cristae morphogenesis and stability. The long isoform of OPA1 was slightly decreased in *Taz* cKO hearts [15]. Thus far, there is no report showing the detailed mechanisms by which TAZ and CL remodeling regulate OPA1 and PHB. 

Mitochondria constantly undergo fusion and fission processes to maintain a proper morphology essential for normal mitochondrial functions [81]. This dynamic is controlled by specific proteins, including mitochondrial fusion proteins mitofusin-1 (MFN1), MFN2, and OPA1, as well as mitochondrial fission proteins like fission protein 1 (FIS1) and dynamin-related protein 1 (DRP1). CL has been shown to play a crucial role in mitochondrial fusion and fission [120]. For example, CL binds to the intermembrane space (IMS) domain of OPA1 and induces fusion of the IMM [121,122]. It also has been shown that CL is necessary for the dimerization and the GTPase activity of OPA1 [123]. Further, mitochondrial fission protein DRP1 shows a strong affinity to CL, which enhances the oligomerization and GTP hydrolysis [124,125]. Although no study directly analyzes how mitochondria fusion and fission changes in BTHS hearts, in *Taz* cKO hearts, MFN1 and MFN2 were dramatically decreased, whereas the long isoform of Opa1 was slightly decreased in cKO hearts compared with control hearts [15]. However, the mitochondrial fission protein Drp1 and its phosphorylation status were unaffected [15]. Deletion of both MFN1 and MFN2 in adult cardiomyocytes results in a decreased size but increased number of mitochondria [126], potentially explaining the observation of smaller but more abundant mitochondria in *Taz* cKO hearts. However, it remains unknown how these fusion and fission proteins in BTHS hearts alter mitochondrial function and contribute to the BTHS cardiomyopathy phenotype. 

CL externalization from the IMM to the OMM has been shown to act as an elimination signal for mitophagy, selective autophagy mediating the degradation of dysfunctional mitochondria [127]. CL interacts with LC3 [127,128], the mammalian ortholog of *Atg8*, and Beclin 1 [129], a central regulator in the formation of autophagosome. KD of CL synthase (CRLS1) and phospholipid scramblase-3 (PLS3), an enzyme responsible for the CL externalization, caused reduced mitophagy in cultured primary neurons [127]. The initiation of mitophagy, but not overall autophagic processes, is impaired in Taz KD mouse embryonic fibroblasts (MEFs) [130]. However, in Taz KD mouse hearts, mitochondria were observed to undergo mitophagy [54]. Thus far, mitophagy has not been monitored in the hearts of BTHS patients. 

## 6. Translating the Basic Research: Potential Approaches to Treat BTHS Cardiomyopathy

The increasing life expectancy associated with BTHS in recent years [13,42] emphasizes the importance of early diagnosis and disease management. It also points to the critical need for the development of therapeutic approaches that will improve the quality of life and daily functioning of BTHS patients. The challenges of developing BTHS therapy include limited patient access, extraordinary phenotypic variability between different patients, and unpredictable clinical presentation [75]. Moreover, until recently, a *Taz* KO mouse capable of recapitulating clinical features of the human condition did not exist [53]. Thus, previous BTHS research was significantly hindered from discovering new targets and testing therapeutic approaches. Current treatments of BTHS focus on multidisciplinary approaches to manage the symptoms from organ-specific manifestations (reviewed in detail in [131]). Surviving BTHS adults display impaired but stabilized cardiac function [42]. Treatment for BTHS cardiomyopathy includes the use of standard heart failure medications, such as angiotensin-converting enzyme inhibitors, angiotensin receptor blockers, β-adrenergic blockers, and diuretics [42,131]. In the setting of LVNC and/or arrhythmia, low-dose anticoagulation may be considered [42]. When heart failure deteriorates and fails to respond to the treatments, patients could be subjected to heart transplantation [42]. It is estimated that heart transplantation has been performed in 14% of BTHS patients [42]. Thus far, there is currently no curative therapy specifically targeting the primary molecular defects in BTHS-related cardiomyopathy. However, multiple therapeutic approaches are under development, some of which are at clinical trials or preclinical stages. These approaches target BTHS cardiomyopathy from different perspectives including targeting mitochondrial dysfunction, restoring *TAZ* gene or protein, and regaining CL biosynthesis (Table 2). Here, we will discuss the molecular basis of each approach, as well as their recent progress and/or challenges. 

### 6.1. Targeting Mitochondrial Dysfunction

Mitochondrial dysfunction has been recognized in BTHS cardiomyopathy since it was first described [48]. Improving mitochondrial function is proposed as an effective approach to ameliorate BTHS cardiomyopathy. Bezafibrate [55,132] and elamipretide [135,136], each of which targets distinct aspects of mitochondrial metabolism, are currently under clinical trial for BTHS therapy. 

Bezafibrate is a pan peroxisome proliferator-activated receptors (PPARs) agonist [133]. Bezafibrate was originally developed as a lipid-lowering drug with a good safety record for long-term use [134]. Bezafibrate activates PPAR/PPAR γ coactivator 1-α (PGC1-α) signaling and promotes transcriptional activation of genes involved in oxidative metabolism [133]. Bezafibrate also increases mitochondrial biogenesis in multiple models [133]. The pharmacological effect of bezafibrate has been exploited to treat heart disease and mitochondrial disorder in patients and animal models [145]. In *Taz* KD mice, bezafibrate treatment ameliorated cardiac dysfunction [146] and DCM induced by isoproterenol [55]. Intriguingly, bezafibrate has been shown to protect CL from degradation and decrease the MLCL-to-CL ratio due to the improvement of mitochondrial function in human BTHS lymphocytes [39]. However, this effect cannot be proved in mouse [55] or patient [132]. The CARDIOlipin MANipulation (CARDIOMAN) study is a UK single-center, double-blinded, randomized, placebo-controlled crossover clinical trial investigating the efficacy of bezafibrate in participants with BTHS [132]. Results from CARDIOMAN revealed no statistically significant improvements in the quality of life in BTHS patients or the primary outcome measurements. Echocardiography data suggested that heart chamber sizes improved but the differences were not statistically significant. The tendency towards improvement was not confirmed in MRI studies performed in parallel. No other cardiac assessments showed significant changes. The small patient number (only 12 participants) makes it challenging to interpret the results and achieve statistical significance. The investigators also noted that most participants continued to participate in an open-label extension study, during which all received bezafibrate (no control group). Over the 60 weeks of the extension study, improvements were seen in the six-minute walk test, muscle function, and fatigue, suggesting that it could take much longer than the four months of therapy used in CARDIOMAN to achieve maximum improvement in mitochondrial function. The major results of the CARDIOMAN trial have been recently released on the ISRCTN registry: https://www.isrctn.com/ISRCTN58006579, accessed on 10 December 2021 and on the BSF website: https://www.barthsyndrome.org/research/clinicaltrials/cardioman.html, accessed on 10 January 2022. 

Elamipretide (also called SS-31) is a water-soluble, aromatic-cationic, mitochondria-targeting tetrapeptide developed by Stealth BioTherapeutics. Elamipretide can readily penetrate and localize to the IMM where it associates with CL. Elamipretide significantly improves mitochondrial functions, including increasing IMM stability, enhancing ATP production, and reducing excessive ROS production in multiple organs and tissues (reviewed in detail in [137]). Elamipretide has been shown to rapidly improve mitochondrial bioenergetics and morphology in BTHS [71], as well as other mitochondrial cardiomyopathy [138] in patient-derived iPSC-derived cardiomyocytes. TAZPOWER is a randomized, double-blind, placebo-controlled, crossover trial of elamipretide followed by a second phase of long-term, open-label treatment extension in patients with genetically confirmed BTHS [135,137]. There were no statistically significant improvements in primary and secondary study endpoints after the phase 1 study (12 weeks of elamipretide treatment) compared to placebo. However, improvements in the 6-min walk test (6MWT), knee extensor strength, the Patient‘s Global Impression of Severity, and the indexed LV stroke volume, showed up in the open-label extension at 36 weeks after completion of the second phase treatment [135,137]. Notably, elamipretide treatment resulted in a significant improvement in average left ventricular stroke volume [135,137].

Besides bezafibrate and elamipretide targeting mitochondrial dysfunction, recent studies in BTHS iPSC and *Taz* cKO mice identified a ROS-CaMKII-RYR2 axis that was responsible for the dysregulated Ca^2+^ handling and cardiomyocyte contractility [69]. ROS scavenger MitoTEMPO and CaMKII inhibitor AIP partially rescued the Ca^2+^ handling in BTHS iPSC-derived cardiomyocytes and *Taz* KO cardiomyocytes [69,71], pointing out that CaMKII inhibitor or ROS scavenger could be a potential invention for BTHS cardiomyopathy. Additionally, endurance training provides beneficial effects on improving mitochondrial function, such as increasing mitochondrial content, decreasing ROS production, and restoring complex II activity in cardiac muscle [64]. 

### 6.2. Restoring TAZ in BTHS

Mutations in *TAZ* result in the complete absence or decreased levels of TAZ protein, or loss of TAZ protein function, causing BTHS [2,3,20]. Restoring TAZ in BTHS patients is a straightforward treatment method. Currently, two approaches aiming to restore TAZ in BTHS are under investigation: gene therapy based on adeno-associated virus (AAV) vector-mediated gene delivery [14,139] and enzyme replacement therapy using recombinant human TAZ fused to a cell-penetrating peptide [131]. CRISPR/Cas9 and other gene-editing technology provide a second opportunity for gene therapy to correct inherited genetic mutations causing disease [147]. However, each BTHS individual may have a different and distinct mutation in their *TAZ* genes [4], requiring gene editing to handcraft a specific pharmaceutical for each individual. Moreover, several technical and ethical considerations must be addressed for safe and efficient clinical translation [147]. Therefore, current investigations still focus on gene replacement therapy and enzyme replacement therapy.

Adeno-associated virus (AAV) vectors are the leading platform for gene delivery in the treatment of a variety of human diseases [148]. Among different AAV serotypes, AAV9 has the best viral genome distribution and the highest protein levels. Moreover, AAV9 displays robust transduction in heart and skeletal muscle [148], two major affected organs in BTHS, making it suitable for restoring TAZ in BTHS. Proof of concept of AAV9-mediated *TAZ* gene replacement has been tested in *Taz* KD [139] and KO [14] mouse models. Pacak et al. constructed a self-complementary AAV9 that expressed TAZ under control of the desmin promoter (scAAV-Des-TAZ) [139]. Administration of scAAV-Des-TAZ to *Taz* KD mice improved, but did not fully normalize, cardiac function [139]. Wang et al. recently applied standard or self-complementary AAV9 that expressed full-length human TAZ under the synthetic CAG promoter (AAV-CAG-TAZ) [14]. In *Taz* gKO mice, AAV-CAG-TAZ rescued perinatal lethality and returned cardiac function to normal. However, at 4 months of age, treated mice exhibited declining heart function. In *Taz* cKO mice, ~70% transduction of adult cardiomyocytes prevented cardiac dysfunction for over 4 months and was able to reverse mild established cardiac dysfunction [14]. However, a lower dose that transduced ~30% of cardiomyocytes had a more variable and less durable effect, suggesting that transduction of a large majority of muscle cells is required for durable efficacy [14]. According to the BSF website, the details of a gene replacement therapy clinical trial for BTHS are developed and regulatory approval will be sought. The enrollment may be 1.5 to 2 years.

Enzyme replacement therapy (ERT) is a treatment whereby replacement enzymes are given to patients who suffer from enzyme deficiencies or malfunction. Recombinant TAZ proteins have been engineered to contain cell-penetrating peptides (CPPs), either alone or in combination with endosomal escape peptides (EEPs), for enzyme replacement therapeutics in BTHS [131]. The effect of recombinant TAZ enzyme replacement therapy has not been published yet. In a conference abstract, recombinant human TAZ fused to a cell-penetrating peptide (hTAZ-CTP) was reported to successfully correct the MLCL/CL ratio in *Taz* cKO hearts and promote some improvements in cardiac performance. 

### 6.3. Targeting CL Biosynthesis

In BTHS, TAZ deficiency causes inefficient remodeling of CL, resulting in decreased levels of nascent and mature CL and increased MLCL, leading to elevated ratios of MLCL to total CL [7,8,9,10,11,21,149]. Manipulation of the CL biosynthesis pathway (Figure 3) to overcome TAZ deficiency may rescue abnormal CL metabolism and thus prevent or rescue BTHS manifestation. 

Due to the lack of acyl specificity in de novo CL synthesis, the composition of acyl chains in nascent CL largely depends on concentrations of specific free fatty acids [22]. Studies in cultured cells suggest that linoleic acid (LA) supplementation restores CL levels and normalizes MLCL/CL ratios, likely due to increased incorporation of linoleoyl groups into nascent CL resulting in the production of mature CL without requiring the remodeling process [140]. This phenomenon has been further validated in BTHS patient iPSC-derived CMs, where cardiomyocyte contractility was significantly rescued by LA treatment [71], suggesting that LA supplementation could be a potential therapeutic approach for BTHS cardiomyopathy. 

PLA2 initiates remodeling of nascent CL by catalyzing its deacylation to MLCL [22]. In BTHS, where reacylation of MLCL is impaired, MLCL accumulates [22]. Deletion of the CL-specific phospholipase Cld1 in yeast rescued *Taz* mutant phenotypes, including respiratory and fermentative growth defects, lifespan, and mitochondrial morphology and function [78,150]. Due to their mitochondrial membrane localization, calcium-independent phospholipases iPLA2b and iPLA2γ have been suggested to play major roles in the deacylation of nascent CL [142,143,151]. In *Taz* mutant Drosophila, iPLA2β KO partially restores MLCL/CL ratios and rescues phenotypic features [74]. In Taz KD mice, iPLA2γ KO partially rescues abnormal CL profiles [76], suggesting that inhibition of iPLA2γ alone could not rescue phenotypes caused by TAZ deficiency. Bromoenol lactone (BEL) is a suicide iPLA2 substrate that selectively targets both iPLA2b and iPLA2g, but not other members of the PLA2 family, in an irreversible and dose-dependent manner [144]. BEL treatment restored MLCL/CL ratios in *Taz* mutant Drosophila [74], BTHS lymphoblasts [74], and BTHS iPSC-derived cardiomyocytes [71]. Thus, BEL treatment would be expected to block the generation of MLCL from nascent CL, ameliorating the increased MLCL/CL ratios and cardiomyopathy observed in BTHS.

Thus far, neither LA supplementation nor BEL treatment has been studied in an in vivo mammalian model of BTHS cardiomyopathy. Recently, Elkes et al. isolated soleus muscle from Taz KD and control mice that were under dietary LA supplementation and performed ex vivo analysis for contractile function. The results revealed that LA supplementation ameliorated the soleus contractile function impairment in Taz KD mice [141]. Further in vivo analysis utilizing *Taz* KO mouse models is required for testing the therapeutic effects of LA supplementation and BEL, as well as determining their proper doses and potential side effects. Moreover, the combined treatment of LA and BEL could generate an increase of mature CL and reduce MLCL, working synergistically to overcome the effects of TAZ deficiency. However, it will be challenging to translate these two agents into clinical usage and treat BTHS patients because currently neither LA nor BEL have been used in a human setting. Extensive preclinical studies that yield preliminary efficacy, toxicity, pharmacokinetics, and safety information are essential for moving the preclinical proof-of-concept studies to clinical development. 

## 7. Summary and Perspectives

BTHS cardiomyopathy is a unique pediatric cardiomyopathy caused by mutations in *Taz* that lead to abnormalities in CL [2,3,20]. BTHS cardiomyopathy exhibits key features of other inherited mitochondrial cardiomyopathies, but also has unique features, including onion-shaped mitochondria and RCS disorganization [1,48]. BTHS cardiomyopathy is life-threatening in infants and youth, but stabilizes in adults. Both clinical and basic research studies have been carried out to uncover the etiology and molecular basis of BTHS cardiomyopathy, with a goal of yielding therapeutic targets. Findings from studies of BTHS cardiomyopathy will delineate the detailed roles of TAZ and CL in mitochondrial function in the heart and advance our knowledge of the molecular basis of mitochondrial cardiomyopathies.

Despite major progress toward understanding BTHS cardiomyopathy, there are still outstanding questions that will require extensive multidisciplinary investigation. CL is an essential lipid of mitochondria, an important subcellular organelle throughout all tissues, but cardiac and skeletal muscle, and neutrophils, are the predominantly affected tissues in BTHS. The requirement for TAZ in different organs/cells and the underlying molecular etiology of BTHS remain to be tested. It is also puzzling why/how BTHS cardiomyopathy becomes stabilized in adults. Understanding the mechanism by which BTHS cardiomyopathy becomes stable will help to manage BTHS cardiomyopathy at different life stages and identify potential approaches to favor stabilization at earlier stages. Recent studies revealed that *Taz* cKO mice display DCM with impaired but stable fractional shortening, consistent with surviving BTHS patients [152], suggesting that specific mechanisms are involved in the stabilization of BTHS cardiomyopathy. The stage-specific roles of TAZ and CL in cardiomyocytes remain largely unknown. Detailed time course studies of mitochondrial functional and morphological analysis in *Taz* cKO hearts will be important to understand how the cardiomyopathy becomes stable. Moreover, temporal deletion of TAZ in cardiomyocytes at different stages will help to understand requirements for TAZ at different stages of cardiomyocyte development. In addition, it is unknown whether other mammalian acyltransferases, such as acyl-CoA:lysocardiolipin acyltransferase 1 (ALCAT1) [40] or monolysocardiolipin acyltransferase 1 (MLCLAT1) [41], compensate for the effects of TAZ deficiency in adult cardiomyocytes. There are multiple abnormalities in CL profiles in BTHS, including low CL concentration, abnormal CL fatty acyl composition, and elevated MLCL to CL ratios [7,8,9,10,11,18,21,149], making it challenging to dissect which of these individually or combinatorially contribute to molecular defects. Moreover, it remains unclear whether TAZ may also have CL-independent functions. Further intensive investigations will help us to address these unanswered questions concerning the etiology of BTHS cardiomyopathy and yield improved therapeutic approaches.

## Figures and Tables

**Table 1:** Experimental models of BTHS cardiomyopathy. Summary of the BTHS experimental models with cardiomyopathy phenotype, including *Taz* knockdown (KD)/knockout (KO) murine models, mouse embryonic stem cell (ESC)-derived cardiomyocytes, and human-induced pluripotent stem cell (iPSC)-derived cardiomyocytes. DOX: doxycycline; mo.: month.

*Taz* Knockdown Murine Models					
DOX Induction	DOX Condition	Knockdown Efficiency	CL Abnormalities	Cardiac Mitochondrial Phenotype	Cardiac Function Phenotype
**Gestation**	625 mg/kg chow [54,55,56,57,58,59,60,61,62,63]	>90% mRNA reduction [54,55,57–59] >95% protein reduction [54,59]	Elevated MLCL and MLCL/CL ratio [54,55,57]. Decreased mature CL [57,59]. Total CL reduction [54,55,56,58,59,60].	Prior to cardiac dysfunction: increased mitochondrial number [54]; mitochondrial aggregation [54]; abnormal morphology (onion- and string-shaped mitochondria, abnormal cristae) [54,56]; decreased respiration capacity [57,58,59,61,64]; increased ROS [47,59]; decreased mitochondrial Ca^2+^ uptake [63].	Normal heart function at 2 and 5 mo. [55,56,57,59]. Dilated LV chamber and systolic dysfunction at 7–8 mo. [54].
	200 mg/kg chow [65,66]	~90% mRNA reduction [65]	Elevated MLCL [65]. Decreased mature CL [65].	Increased mitochondrial number [65,66]; mitochondrial aggregation [65,66]; giant or smaller mitochondria [66]; abnormal cristae [66]; decreased ETC complex I, II, or III activity at 2 and 5 mo. [66].	Systolic dysfunction at 5 [64,66] and 7 mo. [65].
	2 mg DOX/mL water [67]	70–80% mRNA reduction [67]	Elevated MLCL/CL ratio [67].	Decreased mitochondrial number [67]; giant or smaller mitochondria [67], and abnormal cristae at E13.5 [67]. Decreased mitochondrial density [67], vacuolated cristae [67], smaller mitochondria at newborn stage [67].	Prenatal and perinatal death [67]; Noncompaction cardiomyopathy [67]; and defective ventricular septation at E13.5 [67].
**Adult age**	625 mg/kg chow (2–4 mo.) [68]	80% mRNA reduction in the induction period [68]	Decreased mature CL [68].	Normal OXPHOS activity but increased ROS production at 4 mo. [68].	N/A
	2 mg/mL water (3–8 mo.) [67]	N/A	Elevated MLCL Decreased total CL [67].	N/A	N/A
***Taz* Knockout Murine Models**					
	**Knockout Strategy**	**Knockout Efficiency**	**CL Abnormalities**	**Cardiac Mitochondria Phenotype**	**Cardiac Function Phenotype**
**Global knockout**	Taz germline deletion [14].	Taz protein absence in heart tissue [14].	Elevated MLCL/CL ratio [14].	Mitochondrial aggregation [14]; increased mitochondrial number [14]; smaller mitochondria [14]; abnormal cristae [14].	20% of gKO mice survive postnatally [14]. Survivors displayed heart failure with cardiomyocyte apoptosis and cardiac fibrosis starting at 3 mo. [14].
**Cardiac- specific knockout**	Cardiomyocyte-specific Cre (*Myh6*-Cre [14,69] or *Xmlc2*-Cre) [15].	Taz protein decreased in heart tissue, isolated CM and isolated cardiac mitochondria [14,15].	Elevated MLCL and MLCL/CL ratio [14,15]; decreased total CL and mature CL [15]; increased nascent CL [15]; accumulated CL biosynthesis precursor [15].	Prior to cardiac dysfunction: increased mitochondrial number [15], smaller and longer mitochondria [15]; abnormal shapes (onion- and donut-shaped) [15], disorganized and hyperbranching cristae [15]; impaired mitochondrial respiration [15], elevated ROS [15,69].	cKO:*Xmlc2*-Cre: less than 5% lethality with significantly enlarged hearts, majority developed DCM at 4 mo. without cardiac fibrosis [15]. cKO:*Myh6*-Cre: cardiac dysfunction at 2 mo. [14]. Increased heart weight, CM apoptosis, and cardiac fibrosis at 6 mo. [14]; increased arrythmia vulnerability at 1.5 mo. [69].
**Cultured Cardiomyocytes (CMs)**					
	**Generation of iPSC**	**CL Abnormalities**	**Mitochondrial Phenotype**		**Function**
**ESCs derived CMs**	TAZ KO [70]	Increased MLCL/CL ratio [70]; increased nascent CL [70]	Lost cristae parallel orientation and form branching lamellae cristae ratio [70]		N/A
**iPSC derived CMs**	BTHS patient-derived iPSC [57,71]; TAZ mutant by CRISPR-Cas9 mediated gene editing [71]	Increased MLCL/CL ratio and nascent CL [57,71]	Smaller mitochondria [71]; decreased respiratory capacity and ATP production [57,71]; elevated ROS [57,71].		Abnormal sarcomere structure [57,71]; decreased contractility [71].

**Table 2:** Potential therapeutic approaches of BTHS. BTHS treatments focus on restoring TAZ protein and targeting mitochondrial dysfunction and CL biosynthesis. ROS: reactive oxygen species; RYR: ryanodine receptor; iPLA2: calcium-independent phospholipases A2.

	Therapy	Mechanism	Clinical Trial
**Targeting Mitochondrial Dysfunction**	Bezafibrate	Pan peroxisome proliferator-activated receptors (PPARs) agonist that promotes transcription activation of genes involved in oxidative metabolism and mitochondrial biogenesis [55,132,133,134].	CARDIOlipin MANipulation (CARDIOMAN) [132]
	Elamipretide	Water-soluble, aromatic-cationic, mitochondria-targeting tetrapeptide to improve mitochondrial function [71,135,136,137,138].	TAZPOWER [135,137]
	ROS scavenger or CaMKII inhibitor	Partially rescues Ca^2+^ handling defects in cardiomyocytes by attenuating ROS-triggered RYR phosphorylation [69,71].	N/A
**Restoring *TAZ* in BTHS**	Adeno-associated virus (AAV) Gene Therapy	AAV9 mediated *TAZ* gene delivery [14,139].	N/A
	Enzyme replacement therapy (ERT)	Recombinant human TAZ fused to a cell- penetrating peptide (hTAZ-CTP).	N/A
**Targeting CL Biosynthesis**	Linoleic acid (LA)	Increased incorporation of linoleoyl groups into nascent CL resulting in the production of mature CL without requiring the remodeling process [71,140,141].	N/A
	Bromoenol lactone (BEL)	Inhibition of iPLA2 by BEL blocks initiation of the CL remodeling process, ameliorating the increase in MLCL observed in BTHS [71,74,142,143,144].	N/A

## Data Availability

Not applicable.
